# Complexity Measures in Magnetoencephalography: Measuring "Disorder" in Schizophrenia

**DOI:** 10.1371/journal.pone.0120991

**Published:** 2015-04-17

**Authors:** Matthew J. Brookes, Emma L. Hall, Siân E. Robson, Darren Price, Lena Palaniyappan, Elizabeth B. Liddle, Peter F. Liddle, Stephen E. Robinson, Peter G. Morris

**Affiliations:** 1 Sir Peter Mansfield Magnetic Resonance Centre, School of Physics and Astronomy, University of Nottingham, University Park, Nottingham, United Kingdom; 2 Institute of Mental Health, University of Nottingham Innovation Park, Jubilee Campus, Triumph Road, Nottingham, United Kingdom; 3 National Institutes of Health, Bethesda, Maryland, United States of America; Hospital for Sick Children, CANADA

## Abstract

This paper details a methodology which, when applied to magnetoencephalography (MEG) data, is capable of measuring the spatio-temporal dynamics of ‘disorder’ in the human brain. Our method, which is based upon signal entropy, shows that spatially separate brain regions (or networks) generate temporally independent entropy time-courses. These time-courses are modulated by cognitive tasks, with an increase in local neural processing characterised by localised and transient increases in entropy in the neural signal. We explore the relationship between entropy and the more established time-frequency decomposition methods, which elucidate the temporal evolution of neural oscillations. We observe a direct but complex relationship between entropy and oscillatory amplitude, which suggests that these metrics are complementary. Finally, we provide a demonstration of the clinical utility of our method, using it to shed light on aberrant neurophysiological processing in schizophrenia. We demonstrate significantly increased task induced entropy change in patients (compared to controls) in multiple brain regions, including a cingulo-insula network, bilateral insula cortices and a right fronto-parietal network. These findings demonstrate potential clinical utility for our method and support a recent hypothesis that schizophrenia can be characterised by abnormalities in the salience network (a well characterised distributed network comprising bilateral insula and cingulate cortices).

## Introduction

Electrical brain activity, recorded by magnetoencephalography (MEG) or electroencephalography (EEG), is typically analysed using time-frequency decomposition to reveal a set of oscillatory signals. These signals, termed neural oscillations, are observed across a wide spectral range (~0–200 Hz) and offer unique insights into the electrophysiological mechanisms that underpin brain activity (e.g. [1]). Oscillations reflect rhythmic and synchronous current flow across many cells; evidence suggests that large amplitude alpha (8–13 Hz) and beta (13–30 Hz) oscillations are markers of *inactivity*, since when a cortical network is engaged, the amplitude of these oscillations is reduced (e.g. [2,3]). At a cellular level this makes logical sense; breakdown in synchrony across cells facilitates information encoding within a network: Little information can be encoded in a network of cells firing in synchrony; however a breakdown in synchrony, which allows cells to fire independently, will increase capacity for processing. Thus, loss of synchrony (decrease in oscillations) reflects increased processing. In electrophysiological measurements, a loss of synchrony means measured signals become more disordered in time (i.e. they look more like random noise) and this is often interpreted as a loss of measurable effect. However an increase in disorder likely reflects an increase in processing in the brain and quantification of this process would represent a valuable metric of brain dynamics.

In this paper, we describe a method that enables quantitative assessment of the spatio-temporal dynamics of disorder in the brain. Our method is based upon entropy which, in a broad sense, is often taken as a measurement of disorder; the higher the entropy in a system, the greater the disorder. More specifically, entropy allows assessment of moment-to-moment temporal variability in electrophysiological timecourses. Signals with high variability and low predictability (e.g. Gaussian noise) exhibit high entropy whereas highly ordered signals (e.g. oscillations) exhibit low entropy. In this sense, entropy can be thought of as a measure of how much information is in a system, with high entropy (high disorder) meaning more information. There is a growing literature, reviewed recently by Garrett et al. [4] which shows that measuring disorder (or moment-to-moment variability) provides novel and exciting insights into human brain function in health and disease. Specific examples of the use of entropy or related measurements include neurodevelopment [5,6], autism [7], Alzheimer’s disease [8] and schizophrenia [9] amongst others. These measurements show great promise, however present methodologies are limited; some studies involve MEG or EEG sensor space measures, precluding generation of accurate spatial information. Further, most studies assess entropy across large time windows which precludes assessment of temporal dynamics. In the present paper, we aim to highlight a method that provides: 1) a spatial parcellation of functional brain anatomy based upon entropy measurement and 2) the temporal evolution of entropy throughout a cognitive task. Together this facilitates a unique picture of brain dynamics.

Schizophrenia is a severe and poorly understood neuropsychiatric disorder characterised by a breakdown of thought processes and impaired emotional response. A prevalent theory regarding the neuropathology involves a ‘dysconnection hypothesis’ [10,11], implying abnormal connectivity between spatially separate brain regions. (Note here that we use the generic term ‘connectivity’ to mean either structural (physical connections between brain regions), functional (a statistical interdependence between signals derived from separate regions) or effective (a directed functional interaction between separate regions) connectivity.) Recent work [12] suggests that some of the core symptoms of schizophrenia may be explained by abnormal function in a ‘salience network’, a collection of brain regions which includes bilateral insula and cingulate cortices. fMRI studies show that these regions exhibit a high degree of functional connectivity in healthy individuals and recent work shows abnormal effective connectivity between nodes of the salience network in schizophrenia patients relative to controls [12]. Such abnormalities may have implications for entropy measurement. Fernández et al. [13] summarise an elegant argument that ‘progressive isolation of two brain regions would allow each region to express its own dynamical characteristics. Assuming stationarity of connectivity, this could lead to increased disorder (entropy) in the nodes of brain networks that exhibit a disconnection. This, coupled with the salience network findings summarised above, suggests a hypothesis that *schizophrenia patients would show increased entropy in separate nodes of the salience network*, reflecting the breakdown of connectivity. Previous work [9,14,15,16,17,18,19,20,21] (see also [13] for a review) shows some support for increased disorder in schizophrenia patients relative to controls. However there is disagreement in the literature, with other studies showing decreased disorder (e.g. [22,23,24]). In the present paper, we will use our methodology to measure the spatio-temporal signature of disorder, and provide direct inference on where, and when, the brain of a patient exhibits abnormalities.

In what follows, we describe how to combine a recently developed MEG based entropy measurement [25] with independent component analysis (ICA) to elucidate the spatiotemporal dynamics of disorder in the human brain. We use MEG since it offers the best compromise of non-invasive measurement with good (~5 mm) spatial resolution and excellent (~1 ms) temporal resolution. We apply our method in healthy control subjects, and schizophrenia patients, with both groups undertaking the same two cognitive tasks. In healthy controls we use the spatiotemporal dynamics of entropy to build a unique picture of brain activity, and to test the hypothesis that increased cortical processing leads to *localised* and *transient* changes in disorder. We further show how, in healthy controls, these entropic dynamics relate to the more classical oscillatory measures. In schizophrenia, we show that our methodology has clinical utility by testing directly our hypothesis that dysconnectivity within the salience network will manifest as increased disorder in the cingulo-insula cortices. Finally, we compare our measurements (which are based upon rank vector entropy (RVE)) with a more established measure of entropy called multi-scale entropy [26,27].

## Methods

MEG data were recorded in 14 healthy volunteers and 13 patients satisfying the DSM-IV criteria for schizophrenia. Nine of the patients were receiving anti-psychotic medication, but none had experienced a change in antipsychotic, antidepressant, or mood-stabilizing medications in the 6 weeks prior to the study. A clinical interview by a research psychiatrist was performed to ensure that the controls were free from a current psychiatric disorder, history of psychotic or neurological disorders, or a history of psychotic illness in a first degree relative. The study was given ethical approval by the National Research Ethics Committee, Derbyshire, United Kingdom and the University of Nottingham Medical School Research Ethics Committee. All participants gave written informed consent.

### Cognitive tasks

All subjects completed two cognitive paradigms; a visual Sternberg and a relevance modulation (RM) task.

#### Sternberg

The Sternberg task allows measurement of brain activity during the encoding, maintenance and retrieval phases of working memory. Here, we used a visual Sternberg experiment (as described in [28]). A single trial comprised presentation of two *example* visual stimuli (black abstract shapes on a grey background, shown for 600ms with 1s between onsets). This was followed by a 6s maintenance period and a third *probe* stimulus (duration 3s). The subject was asked to respond if the *probe* matched either of the two *example* stimuli. A single block comprised three trials followed by a rest block of 36s (from which we calculate baseline entropy and oscillatory amplitude); 15 blocks were presented to each subject; the probability that a probe matched one of the examples in any given trial was 0.5.

#### RM task

In the RM task, the task-relevance of visual stimuli was manipulated [28]. Subjects were shown a series of visual stimuli (of duration 800ms) in which pictures of butterflies were alternated with pictures of ladybirds. Inter-stimulus-intervals were jittered, with an average of 1.92 ± 0.08s. A single block comprised presentation of 40 alternating stimuli, followed by a resting phase (from which baseline entropy and oscillatory amplitude were computed). Blocks were 120s in total. At the beginning of each block, subjects were informed whether the relevant stimuli were butterflies or ladybirds. In blocks where butterflies were relevant (BF), the subject’s task was to respond when the butterfly on the screen matched an example butterfly (shown at the start of the block). Matches were identified based on shape, outer wing colour and inner wing colour. In blocks where ladybirds were relevant (LB), the task was to respond if the number of red ladybirds was equal to the number of yellow ladybirds. In both block types, the target stimuli were rare (probability = 0.05), and these trials were excluded from subsequent analyses. Eight blocks were presented to each subject, in the order: BF, LB, LB, BF, LB, BF, BF, LB.

All participants were given an explanation of the two tasks outside the scanner as well as an opportunity to practice, ensuring all subjects could complete both tasks.

### Data Acquisition

MEG data were acquired using the third order synthetic gradiometer configuration of a 275 channel MEG system (MISL, Coquitlam, Canada), at a sampling rate of 600Hz and using a 150Hz low pass anti-aliasing filter. Three fiducial coils were attached to the participant’s head at the nasion, left and right preauricular points. These coils were energised periodically to allow localisation of the head relative to the geometry of the MEG sensor array. Prior to data acquisition, the surface of the participant’s head was digitised (Polhemus, Isotrack). Subsequent surface matching of the digitised head shape to a head shape extracted from an anatomical magnetic resonance (MR) image allowed coregistration of brain anatomy to MEG sensor geometry. The anatomical MR image (1 mm cubic resolution) was acquired using a Philips Achieva 3T MRI system running an MPRAGE sequence.

### Data Analyses

**Fig 1:**
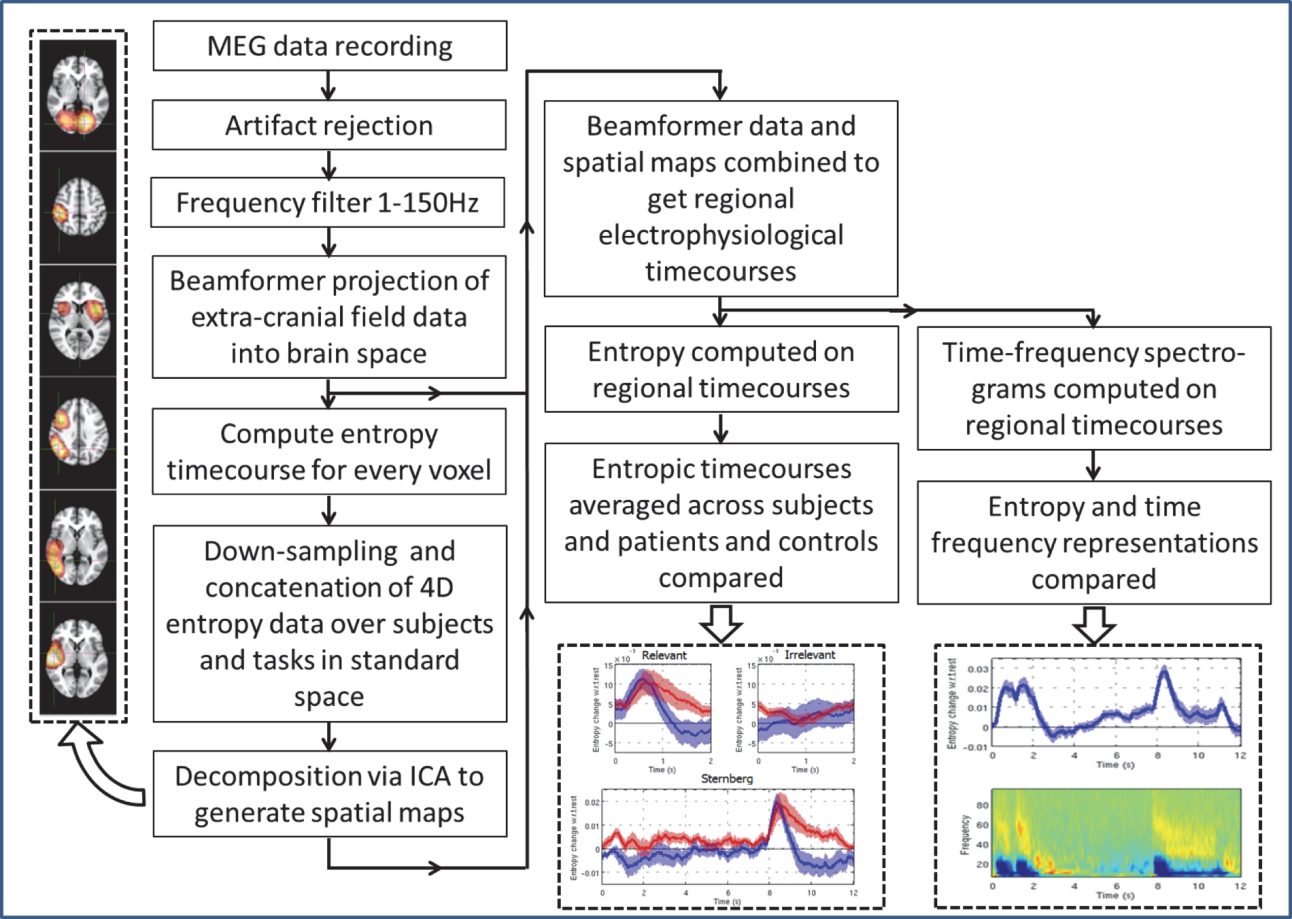
Flow chart summarising the overall data analysis pipeline.

MEG data were initially inspected for artefacts. Trials containing excessive interference were removed. Participants found to have moved more than 8 mm during the scan were rejected from the study. This initial data screening left 11 healthy controls and 11 patients, who were used in subsequent analyses. Data analysis comprised multiple stages; the overall pipeline is summarised by Fig 1, and each component part is described in detail below.

### Beamforming

Beamforming [29,30,31,32,33] was used to project extra-cranial MEG data into source space to generate timecourses of electrophysiological activity at a set of locations (voxels) in the brain. The electrical source strength, ${\hat{Q}}_{\theta }\left(t\right)$, at location and orientation (**θ**), and at time *t*, was estimated using a weighted sum of sensor measurements thus:

$${\hat{Q}}_{\theta }\left(t\right)=w_{\theta }^{T}m\left(t\right)$$ 1

where **m**(*t*) is a vector of magnetic field measurements made at *M* sensors at time *t* and **w**_**θ**_ is a vector of weighting parameters tuned to location and orientation **θ**. The weights were derived based on minimising the variance of the output timecourse, with a linear constraint that variance originating at **θ** remains. Mathematically, the weights are given by:

$$w_{\theta }^{T}={\left[h_{\theta }^{T}{\left\{C+\mu \Sigma \right\}}^{-1}h_{\theta }\right]}^{-1}h_{\theta }^{T}{\left\{C+\mu \Sigma \right\}}^{-1}$$ 2

where **h**_**θ**_ is the lead field vector for location and orientation **θ**, **C** represents the data covariance matrix and **Σ** = *η* ^2^ **I** where *η* ^2^ represents an estimate of the white noise at each MEG sensor (estimated as the smallest singular value of **C**). **I** is the identity matrix and *μ* is a regularisation parameter (here *μ* = 4). Source orientation at each voxel was computed using a non-linear search for the maximum projected signal to noise ratio; note that source orientation was restricted to the tangential plane.

In the beamformer implementation used, voxels were defined at the vertices of a regular (8 mm) grid spanning the entire brain. The dipole model used to calculate the lead fields was based upon a multiple local spheres head model [34], and the field equation derived by Sarvas [35]. Covariance was computed using data from the entire experiment and spanning the full 1–150 Hz frequency range in order to minimise the effect of correlated sources and maximise reconstruction accuracy [36].

### Rank Vector Entropy (RVE)

**Fig 2:**
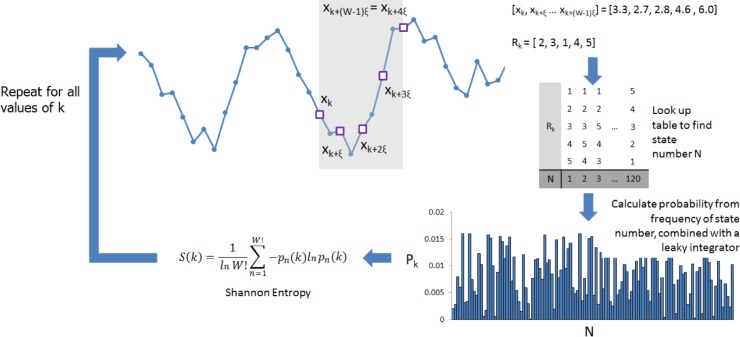
Schematic diagram detailing the calculation of Rank Vector Entropy.

Rank Vector Entropy (RVE) is a computationally efficient and high signal to noise algorithm recently introduced by Robinson et al. [25]. It combines symbolic entropy with a leaky integrator in order to transform timecourses of electrophysiological activity into a timecourse showing the temporal evolution of entropy. Note that RVE is nonparametric and is independent of source amplitude. The procedure is depicted in Fig 2.

Consider a timecourse, **X**, measured at a sampling rate, *f*. We first select *W* samples, at integer (ξ) intervals, from this timecourse, starting at sample *k*. This means that the selected vector can be written as$W_{k}=\left[x_{k},x_{k+\xi },x_{k+2\xi },x_{k+3\xi }\ldots x_{k+\left(W-1\right)\xi }\right]$. (i.e. if ξ = 2, then every second data point is selected). The vector, **W**_**k**_, is then transformed into its associated rank vector, **R**_**k**_, this means that the indices of the components of **W**_**k**_ are ranked, according to the magnitude of those components. For example, the vector **W**_*k*_ = [3.3,2.7,2.8,4.6,6.0] would be transformed to the rank vector **R**_*k*_ = [2,3,1,4,5] (see also Fig 2).

If the rank vectors contain *W* elements, there are *W!* possible rank vectors to choose from; e.g. if W = 5, the set contains 5! = 120 possible rank vectors. We now assume the existence of a look up table which contains each of the possible rank vectors and allows a conversion from any one rank vector, to a ‘symbol’ (e.g. in the example shown in Fig 2, **R**_*k*_ = [1, 2, 3, 4, 5] would be assigned the symbol *N*_*k*_ = *1*; **R**_*k*_ = [1, 2, 3, 5, 4] would be assigned the symbol *N*_*k*_ = *2* and so on). If we allow the window, **W**_**k**_, to translate in time along the timecourse **X,** by incrementing *k*, this will allow iterative computation of multiple realisations of the rank vector, and therefore calculation of multiple symbols (i.e. we will get one new symbol, each time we increment k). The occurrences (frequency) of each symbol can then be counted. This facilitates computation of a frequency distribution, **F**_**k**_, which shows the frequency of occurrence any one symbol.

The purpose of RVE is to generate information on the temporal dynamics of entropy, which is achieved via the use of a leaky integrator. This is designed to integrate a result over time, but allows a small amount of the input to ‘leak’ out, such that, as time is sequentially advanced, the contribution of previous windows is diminished. In RVE this is achieved by an extra modification of the frequency distribution **F**_**k**_ with each iteration, such that **F**_***k***_ **= αF**_***k*–1**_ where alpha is less than 1. This scaling is applied to all values in the frequency distribution *before* the symbol for the current iteration is incremented. The value of the constant, α is determined by a time constant τ such that *α* = *exp*(−1/*fτ*). τ therefore represents the time taken for the counts to decay to a fraction of 1/e times their original value. Note that if *α* = 1, RVE gives the cumulative frequencies over all samples (i.e. all time); however with *α* < 1, the result generates a frequency distribution that changes in time. This can then be used to define the temporal dynamics of entropy. The frequency distribution, **F**_**k**_, is used to generate a probability distribution, **P**_**k**_, by normalising by the total integral of the frequencies across all symbols. For each step, *k*, the “instantaneous” Shannon entropy can then be computed as:

$$S\left(k\right)=\frac{1}{ln\left(w!\right)}\overset{w!}{\underset{n-1}{\sum }}-p_{n}\left(k\right)ln\left(p_{n}\left(k\right)\right)$$ 3

Note that for RVE to work, the time constant τ must be sufficiently long for all of the states in the frequency distribution to be populated. In the present work, we apply RVE with a time constant τ = 0.3s, ξ = 2 and w = 5. The RVE method was applied sequentially and independently to all voxels in source space (i.e. all voxels in the beamformer projected MEG data). This yielded a 4D dataset for each subject showing the temporal evolution of entropy at all locations in the brain.

### Independent Component Analysis (ICA) and regional analysis

Having generated 4D matrices via entropic transformation, we aimed to decompose these into a small number of temporally independent components, depicting voxels which exhibit similar temporal (entropy) profiles. To achieve this, entropy timecourses from the entire experiment for all voxels were down-sampled to a 1s time resolution. This down-sampling acts to reduce the total amount of data, making ICA computationally feasible. Data were then spatially normalised to a template brain in MNI space and concatenated across subjects and tasks, thus generating a single dataset representing all 22 subjects and both tasks. This 4D dataset was reshaped into a 2D matrix, which we term **Λ**, containing only voxels inside the brain; temporal ICA was then applied such that the measurements in **Λ** were defined as linear mixtures of independent signals **S,** thus:

Λ=AS 4

Here, **A** represents a mixing matrix which defines the contribution of each independent signal to each voxel; the columns of **A** thus represent the spatial profiles of each independent component which can be thought of as a spatial basis set.

Temporal ICA was applied using the fastICA algorithm [37] implemented in matlab [research.ics.tkk.fi/ica/fastica]. The method of application was identical to that described in previous papers [28,38], apart from the number of independent components extracted. Pre-whitening was applied, which used a basic principle component analysis (PCA) (based upon eigenvalue decomposition of the projected envelope data covariance matrix) to reduce the dataset to 45 principal components (to improve computational efficiency). Following this, ICA was applied and 40 components extracted. These 40 components were able to explain 84% of the variance in the multi-subject, multi-task concatenated 4-dimensional dataset. Of these 40 components, 12 were selected for further analysis. When applying ICA to experimental data, the question of how to select components of interest is always an issue. In this paper, we followed the same method as used in previous papers [28,38] and components were originally selected based upon visual inspection. Only those components whose spatial map could be readily interpreted were retained; all of the spatial maps identified either single brain regions, or multiple brain regions, comprising well characterised foci or networks (e.g. left motor cortex, visual cortex, fronto-parietal network etc.). A general linear model analysis showed that the 12 components retained explained 54% of the total data variance across all brain voxels (prior to PCA) and 64% of variance the data retained post ICA. The robustness of these components was also tested using a post-hoc Monte Carlo type analysis (see S1 Appendix for details).

Following ICA, electrophysiological timecourses for each of the 12 spatial components were reconstructed via a weighted sum of the original beamformed voxel timecourses (See Fig 1). The weights were generated directly from the columns of the mixing matrix, **A**, which were thresholded to leave only contributions from voxels within the regions of interest. Reconstruction in this way facilitates a single electrophysiological timecourse for each spatial map derived. These timecourses can then be used to generate a regional entropy signal (at the full temporal resolution, rather than the down-sampled (1s) time resolution used for ICA) and also a regional time-frequency spectrogram. Time-frequency spectrograms were generated via Hilbert transformation of band pass filtered data, employing 33 overlapping bands spanning the 1–150Hz range. Entropy and time-frequency spectrograms were computed independently for the Sternberg and RM tasks and results were averaged across task trials. For the Sternberg task, trial averages were computed within a 0 to 12s time window spanning presentation of the two example stimuli, the maintenance phase, and the probe stimulus. For RM, average signals were calculated within 0 to 2s windows following both relevant and irrelevant stimuli, thus facilitating comparison between conditions.

Entropy signals were compared to time frequency spectra via computation of the Pearson correlation coefficient. Correlation was estimated between the entropy timecourse, and the oscillatory envelope which was again calculated within each of the 33 frequency bands studied. In all cases, correlation was computed using unaveraged data, and on each dataset individually. Results were subsequently averaged across datasets and standard deviation assessed. This analysis was undertaken for all 12 of the brain regions studied.

### Multi-scale Sample Entropy (MSE)

The RVE calculations described above were supplemented with the more established technique of multi-scale sample entropy. This was to verify that any changes observed in schizophrenia using RVE were generally indicative of altered entropy in schizophrenia.

**Fig 3:**
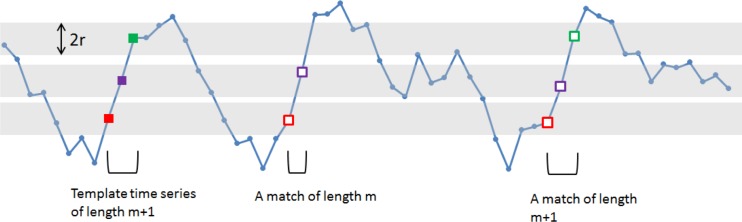
A simulated time-series is shown in blue, the template points are shown in solid squares, and the shaded grey areas indicate the points that are within ± r (tolerance) of these template points. The unfilled squares indicate points that match the template points to form sequences of length m or m+1 (here m = 2). The template points are moved sequentially through the time-series, and the total number of matches of length m, and length m+1 are calculated.

Sample entropy (SampEn) [39] assesses the predictability of a timecourse. Starting at point k, m+1 points are selected as a template time series. Following this, the remainder of the time series is examined for matches to this template (all matches to the first m points, and all m+1 points are counted separately). This analysis is repeated, moving the initial starting point k, and counting the total number of template matches of length m and length m+1. Sample entropy is then defined as the natural logarithm of the ratio of these two numbers, and indicates the likelihood of predicting the subsequent data point from the first m data points in a timecourse (a schematic of this process is shown in Fig 3). There are two variables which affect the estimate of SampEn: m, which governs the length of the template, and the tolerance, r, which governs what qualifies as a match. Here, we employ m = 2 and r = 20% of the timecourse standard deviation [27].

MSE is an extension of sample entropy and calculates SampEn at different temporal scales [26,27]. The scale of the timecourse reflects how much it has been down-sampled. A scale of 1 is the original time series; a scale of 2 indicates that the timeseries has been downsampled by averaging non-overlapping groups of 2 points. Mathematically:

$$Q_{ds}\left(j\right)=\frac{1}{s}\sum_{i=\left(j-1\right)s+1}^{js}Q\left(i\right)$$ 5

where s is the scale factor, *Q* is the input timecourse, and *Q*_*ds*_ is the down sampled version of *Q*. The regional beamformer projected timecourses were used as an input for MSE, in order to allow direct comparison of MSE and RVE. MSE was calculated on a per-trial basis and results averaged across trials, and participants in each group. For the RM task, trials were split into relevant and irrelevant trials and processed independently. The scale factor was varied from 1 to 20 for Sternberg, and from 1 to 10 for RM (due to the shorter data segments).

### Changes in schizophrenia

To test the hypothesis that patients exhibit a greater change in entropy when engaged in a task compared to controls, the measured entropy change due to a task was calculated using both RVE and MSE. For RVE, the average change in entropy across the duration of both the Sternberg and RM tasks was used. For MSE, each scale factor was tested independently. Permutation testing was used to assess statistical significance of the task induced entropy change between patients and controls: 500,000 iterations were performed where two sham data sets were created with an equal mix of patient and control data, to create a null distribution of the difference between two data sets. The real data were compared to this null distribution to assess significance. Bonferroni correction was applied to account for multiple comparisons across the separate regions studied (i.e. threshold for significance, p < (0.05/N), where N = 12). In addition to the permutation test, a non-parametric sum-rank test was also employed, again with multiple comparisons controlled by Bonferroni correction. A difference between groups is reported only if both tests showed statistical significance.

## Results

### The Spatiotemporal signature of complexity

**Fig 4:**
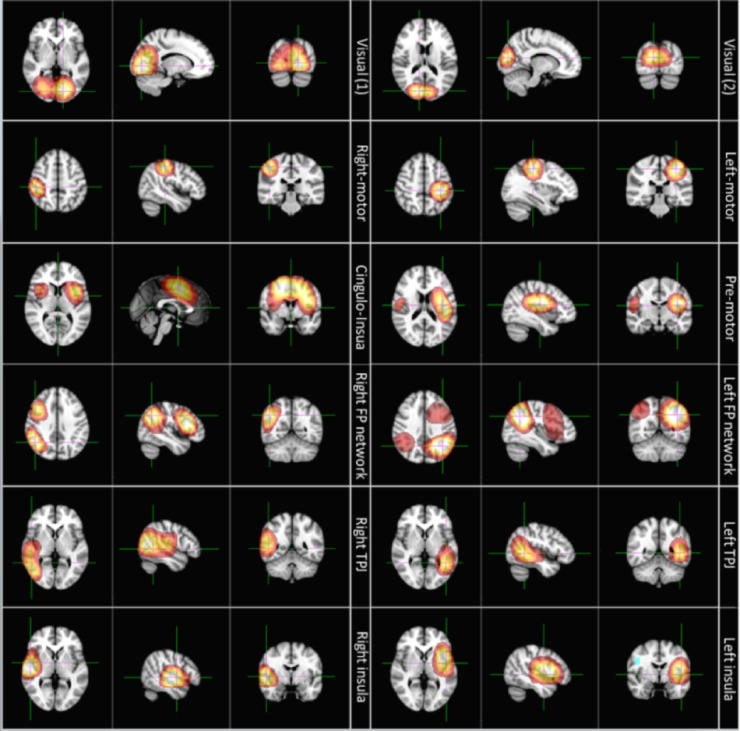
Spatial signatures of 12 independent components, derived from ICA applied to entropic transformation of MEG data concatenated across all subjects, and tasks. Regions identified include the visual cortex, the left and right motor cortices, cingulo-insula cortex, pre-motor cortex, the left and right fronto-parietal networks, the left and right temporo-parietal junction and the left and right insula cortices. This represents a unique method to parcellate the cortex, based upon the temporal signature of entropy. Images are displayed in radiological convention.

The RVE/ICA method yielded a spatial basis set, which is shown in Fig 4. The functional maps depict the spatial signatures of 12 temporally independent components which were selected for further analysis. Each of these maps represents a brain region (or collection of brain regions) that generates an independent temporal evolution of entropy. In this way, we allow spatial parcellation of the cortex, based upon its functional anatomy. The regions selected are well characterised and include visual cortex, motor cortex, cingulo-insula region, pre-motor cortex, the left and right fronto-parietal networks, the tempero-parietal junction and the left and right insula cortices. This spatial basis set was employed as a platform upon which all subsequent analyses were based. It is important to note that this approach offers significant advantages over voxel based metrics. Specifically, ICA allows us to decompose several thousand voxel timecourses into a smaller number of independent components, reducing multiple comparison problems that inevitably arise in subsequent statistical testing (see also discussion).

**Fig 5:**
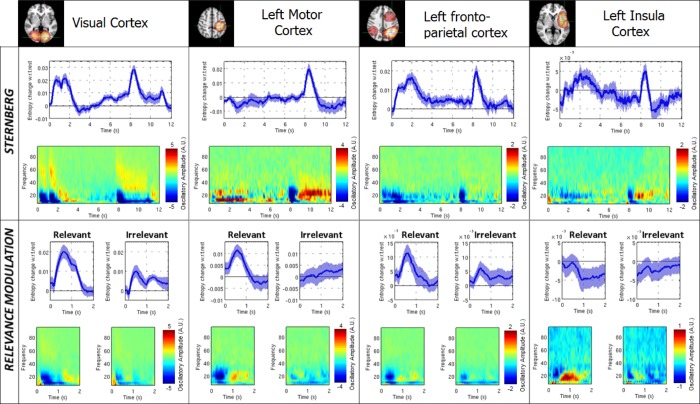
Entropic time-courses and time-frequency spectrograms are given for the visual, left motor, left fronto-parietal and left insula regions. The upper panel shows results for the Sternberg task and the lower panel shows results for the RM task. For entropic timecourses, the blue line shows change in entropy from baseline level; the shaded region shows standard error across subjects. Time-frequency spectrograms show deviation from resting state oscillatory amplitude, with red and blue showing increased and decreased oscillatory amplitude respectively. Note that both tasks elicit transient changes in signal entropy. Note also the differences in temporal profile of entropy across brain regions.

Fig 5 shows entropic time-courses and time-frequency spectrograms for 4 of the 12 regions identified in Fig 4. In the entropy timecourses, the zero-line (black solid line) represents resting level entropy (computed within a rest block), and so timecourses show transient change in entropy induced by the task, relative to baseline. Similarly, the time frequency spectrograms are also plotted relative to baseline, with blue representing decreased oscillatory power with respect to rest and red/yellow representing increased oscillatory power.

Fig 5 shows that both the Sternberg and RM tasks elicit transient, task driven changes in entropy, and neural oscillations. Taking the visual cortex as an example, during the Sternberg task, two peaks in entropy are visible in the first two seconds corresponding to presentation of the two example visual stimuli. A 3^rd^ peak then follows at ~8 seconds corresponding to the probe stimulus. Similar transient increases in entropy are observed for the RM task, with a more prominent effect in the relevant compared to the irrelevant case. Entropy changes during the task differ across brain regions with, for example, the left primary sensori-motor region only demonstrating an appreciable change during the relevant phase of the RM task, and within a ~2s window centred on the probe stimulus during the Sternberg task. Results are presented for the left hemisphere; however similar measurements were made for the homologous regions in the right hemisphere. Interestingly, less marked changes in entropy and time frequency spectrograms were observed in the right hemisphere. The variance in trial averaged entropy was significantly (p<0.05 corrected, non-parametric sum-rank test) lower in the right fronto-parietal network relative to the left, indicating a degree of left hemispheric lateralisation in information processing for both the RM and Sternberg tasks. (The average variance of the entropy timecourse was also significantly lower in the right sensorimotor area relative to the left, as would be expected given that both tasks involved a right handed button press.)

Fig 5 shows the importance of characterising the time evolution of entropy (as distinct from a single measure spanning the whole experiment). Our results are consistent with the notion that increased entropy is associated with a transient increase in local cortical processing. Or put another way, increased information processing necessitates the breakdown of synchrony across neurons to increase efficacy of encoding of information within a local neural network. This manifests as a spatially localised and transient increase in the entropy of the measured electrophysiological signal.

### The relationship between entropy and neural oscillations

**Fig 6:**
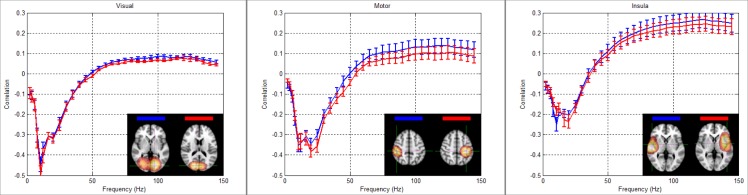
The relationship between neural oscillatory amplitude and signal entropy in the visual, motor and insula cortices. Note the general trend that entropy exhibits a negative correlation with alpha and beta oscillations and a positive correlation with gamma oscillations. Inset images show the spatial maps of the regions used. The mean correlation across subjects is shown and error bars show standard deviation across subjects.

The majority of MEG experiments are processed via exposition of the time-frequency signature of neural oscillations. Comparing entropic timecourses to the spectrograms in Fig 5, it is clear that a relationship exists, with decreases in oscillatory amplitude in the alpha and beta band accompanied by a transient increase in entropy. Given that oscillations and entropy are generated from the same underlying beamformer projected timecourses, coupled with the fact that oscillations are highly predictable and therefore low entropy, this would be expected. However, it proves instructive to probe this relationship further by comparing the time-courses of change in oscillatory amplitude across the frequency spectrum, to the entropic time-courses. Such comparison reveals a non-linear relationship, summarised in Fig 6, which shows temporal correlation between the Hilbert envelope of oscillations and the entropy timecourses. Note that the relationship exhibits a general trend with a negative correlation between entropy and alpha and beta band oscillatory amplitude, and a positive correlation at higher frequency. Note also a weak negative correlation in the delta and theta range. An effect of signal to noise ratio (SNR) is apparent: in general, the SNR of alpha and beta band oscillations is higher than that of gamma oscillations. This probably explains the higher (absolute) correlation between entropy and alpha and beta oscillations, compared to gamma oscillations. Most importantly, we note that that no simple one-to-one relationship exists between the entropic timecourse and oscillatory power in any one frequency band.

Fig 6 also reveals subtle differences in the biphasic response between regions. For example, in the visual cortex, the strongest negative correlation is in the alpha band, whereas the motor cortex demonstrates the strongest negative correlation across both the alpha and beta bands. In insula cortex, the negative relationship also peaks across the alpha and beta bands; however this negative correlation is weaker than in the visual and motor regions, but the insula cortices exhibit a stronger positive correlation between entropy and gamma oscillations. Note also that the zero crossing point differs between regions, being ~50 Hz in the motor and visual cortices but ~40 Hz in the insula cortices. It therefore follows that whilst a relationship exists between entropy and time frequency decomposition, this relationship changes with the brain region studied and entropy cannot be considered a surrogate metric for any one frequency or group of frequencies.

The neural oscillatory amplitudes across all frequencies were combined in a general linear model to probe the degree to which the amplitude envelope of oscillations, across the 1–150Hz band, could explain the entropy observations. The GLM was applied as:

s=Gβ+ε 6

where **s** represents the entropy timecourse. The design matrix, **G**, comprised a single column for every frequency band and contained neural oscillatory envelopes (which were orthogonalised (as required by linear regression) using singular value decomposition of the covariance matrix **GG** ^T^), and **ε** represents the error. The parameters, **β**, represent the contribution of each frequency to the entropy signal and are directly related to the correlation coefficients shown in Fig 6. This procedure, computed individually for 11 healthy controls in the Sternberg data, was used to compute the percent variance explained in the entropy signal, by the neural oscillations. The result showed that, on average over all subjects and all brain regions shown in Fig 4, only 36±1% of the variance in entropy could be explained by oscillations. This simple analysis implies that the entropic signal, whilst clearly showing a significant relationship with oscillations, also contains some unique information that cannot be gained by looking only at time frequency spectrograms.

### Entropic measurement in schizophrenia

**Fig 7:**
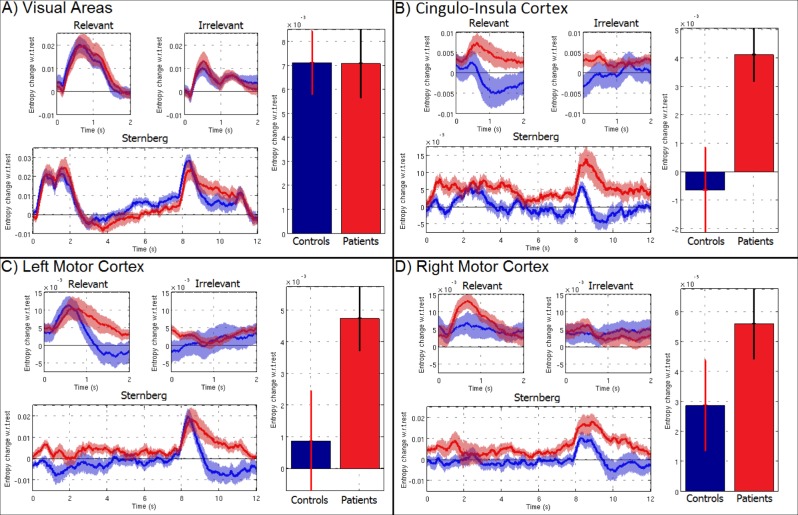
Time-courses in the visual cortex (A), cingulo-insula cortex (B), left motor cortex (C) and right motor cortex (D) showing task induced change in baseline entropy. Results are presented for schizophrenia patients (red) and healthy control subjects (blue) in the relevant and irrelevant phase of the RM task, and the Sternberg task. Bar charts show averaged entropy change during task, compared to rest, collapsed across both tasks. Note that in the cingulo-insula cortex, a significant (corrected p < 0.05) increase in entropy in patients, relative to control subjects is observed. Note also that the difference between patients and controls changes as a function of time, highlighting the importance of dynamic assessment of entropy.

Fig 7 shows trial averaged entropy time-courses, for the Sternberg and RM tasks, for control subjects (blue) and schizophrenia patients (red). Results are shown for the visual cortex (A), the cingulo-insula component (B), the left motor cortex (C) and the right motor cortex (D). As with the entropy metrics in Fig 5, the zero-line represents resting entropy, and time-courses show stimulus induced change from baseline. The bar charts in Fig 7 show mean task induced entropy change in healthy controls (left) and patients (right) for each region; these measurements are collapsed across subjects and tasks (i.e. 22 independent MEG sessions in each group) and error bars show standard error across subjects. The principal result, summarised by Fig 7B, shows that in the cingulo-insula cortex, task induced entropy change is higher in patients than controls. The between group difference was found to be significant at a p<0.05 level, Bonferroni corrected across the 12 brain regions studied (this was true both using permutation testing, and a non-parametric Wilcoxon sum-rank test). In addition to the cingulo-insula component, significantly increased entropy was recorded in both the left and right insula cortices and the right fronto-parietal network. None of the other brain regions in Fig 4 reached statistical significance although a trend was noted in the left motor cortex.

Again note the importance of characterising temporal dynamics of entropy throughout the experiment (as distinct from summary measures collapsed across task/rest windows). Fig 7 shows clear evidence that, whilst a significant increase in entropy in patients is observed throughout the Sternberg and RM tasks, this increase is time dependent. For example, in the RM task, the largest difference between patients and controls occurs following a relevant stimulus, and at approximately 1s latency following stimulus presentation. In the Sternberg task, whilst a difference in entropy between patients and controls persists throughout the average trial, the magnitude of this difference peaks within the 8–10s time window, which coincides with the presentation of the probe stimulus. Other, more subtle trends are noted in the motor cortices. For example, in healthy controls, the entropic response in both the Sternberg and RM tasks is characterised by an entropy increase during stimulation, followed by a decrease below baseline and subsequent recovery to the resting level. Fig 7C shows that, in patients, the increased entropy elicited by the response persists across a larger temporal window, and does not exhibit the post response dip below baseline observed in controls.

**Fig 8:**
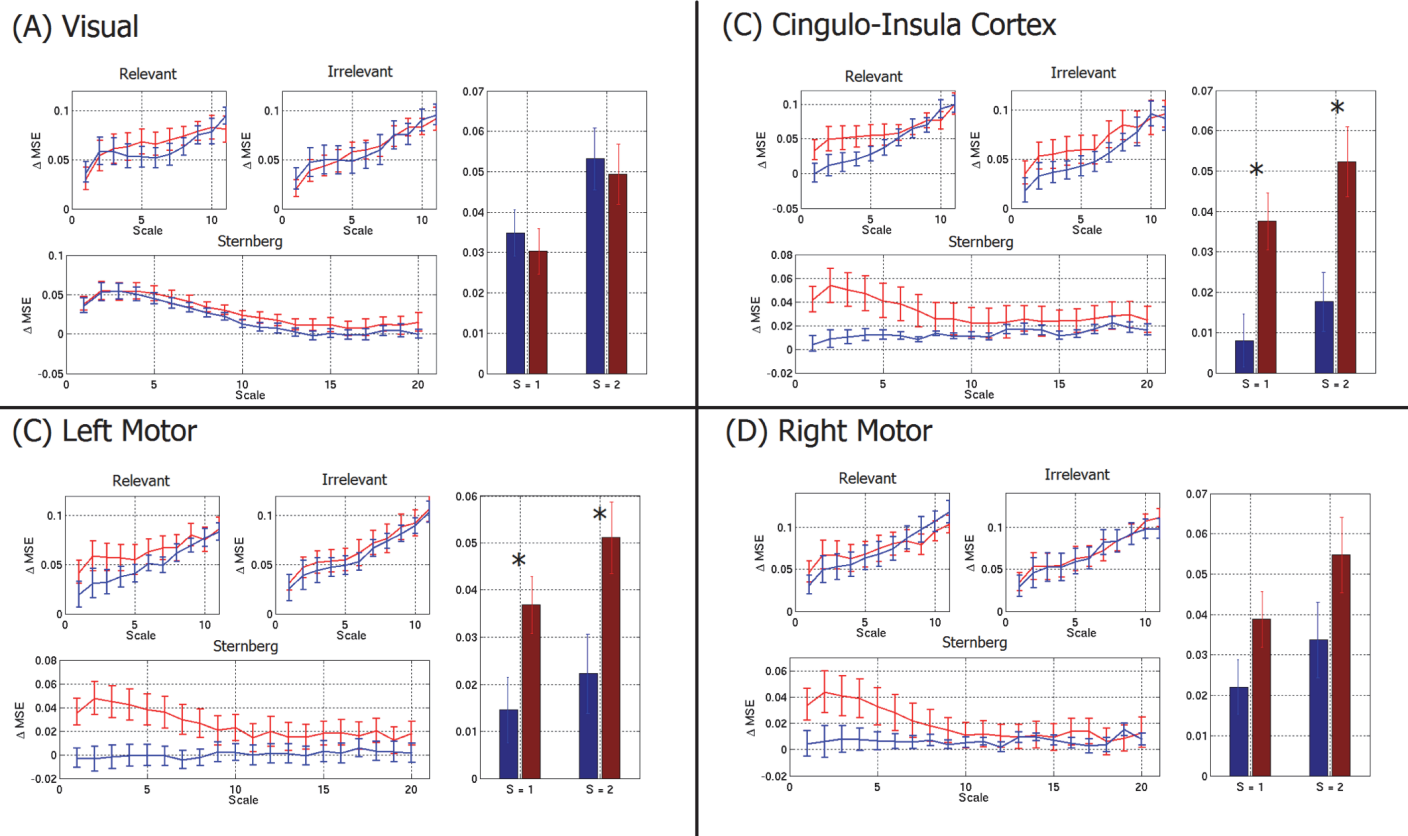
Multi-scale-entropy, measured in the visual cortex (A), cingulo-insula cortex (B), left motor cortex (C) and right motor cortex (D). Graphs show change in MSE from rest in patients (red) and controls (blue) for the RM and Sternberg tasks. Bar charts show specific cases for scales 1 and 2. Note that there is general agreement between RVE (Fig 4) and MSE (Fig 5) in showing an increased entropy difference in patients relative to controls. Note also that this difference depends on temporal scale, and is maximum when entropy is measured on the very short timescale (i.e. scales of 1 and 2). * indicates p<0.05 corrected.

The difference in entropy between patients and controls, observed in Fig 7, is based upon RVE, which allows direct inference on the temporal evolution of entropy in the brain. In this RVE implementation, we assess temporal variation on a timescale of 3.4ms (taking every second time sample at a sample rate of 600Hz); entropy is calculated using a leaky integrator with a (1/e) window width of 0.3s. In other words, whilst the timecourses in Figs 5 and 7 look temporally smooth, RVE assesses temporal variability at a millisecond timescale. This said, entropy can be measured at any temporal scale and it proves instructive to assess multiple scales in order to test whether the observed differences persist when using a slower timeframe. To achieve this, we used MSE [26,27], which has been employed in previous schizophrenia measurements (e.g. [9]) and in addition to offering a means to assess the effect of temporal scale, also allows a means to compare our RVE methodology to others previously employed. Results are given in Fig 8, which shows the task induced change in MSE (ΔMSE) plotted against temporal scale in the visual cortex (A), cingulo-insula cortex (B), left motor cortex (C) and right motor cortex (D). Results are in agreement with our RVE metrics (Fig 7) in showing significantly increased task related entropy change in patients compared to controls. (p<0.05, corrected for multiple comparisons across the 12 regions). More importantly, Fig 8 also shows clear evidence that this observation is critically dependent on the timescale at which entropy is assessed, with significant results observed only at the most rapid timescales (scales of 1 and 2, corresponding to sampling at 1.7ms and 3.4ms scale respectively). These timescales are approximately equivalent to those used for RVE. Finally, note that whilst the primary results showing increased entropy in patients relative to controls is confirmed by MSE, no information on the temporal evolution of entropy is generated.

## Discussion

### Methodology and the relationship between entropy and neural oscillations

Our aim in this methods paper was to elucidate spatial variability and temporal dynamics of disorder in MEG signals. RVE [25] combines symbolic entropy with a leaky integrator in order to characterise the temporal evolution of entropy. This facilitates non-linear transformation of beamformer projected MEG data into timecourses showing ‘instantaneous’ entropy of the localised signal. Note that this analysis was applied to broad-band (1–150 Hz) signals as narrow-band signals have low entropy by definition. Beamforming and entropic transformation yields a set of temporal signals (one per source space voxel) which, using our method, are concatenated across subjects and decomposed into a smaller set of independent components. This decomposition has the key advantage that it reduces data dimensionality: Reducing several thousand voxels to a small number of independent components reduces the multiple comparisons problem that will inevitably be faced in subsequent statistical testing. Without ICA, one would have to correct for multiple comparisons across voxels; since Bonferroni correction would prove overly conservative (due to the inherent spatial smoothness of the beamformer reconstruction) this would necessitate estimation of spatial smoothness using techniques such as Gaussian Random Field Theory [40]. Using ICA, the timecourse data are not only reduced in dimension, but the method naturally generates an ICA mixing matrix which provides an effective spatial map, showing the contribution of each voxel to each independent component. Following decomposition, multiple comparisons can be corrected accurately using the Bonferroni method, since the individual regions represent independent measures of brain activity. The ICA mixing matrix also provides a principled way to compute a weighted average of entropy signals within the derived regions of interest. Averaging timecourses across multiple voxels has the desirable effect of improving signal to noise ratio, meaning that the regional entropy timecourses are likely to exhibit higher signal-to-noise ratio than underlying voxel timecourses, again demonstrating the utility of ICA. Note that, using MEG, the natural smoothness of the beamformer reconstruction means that this increase in SNR is not as pronounced as it would be in, for example, fMRI where the increased spatial resolution means that neighbouring voxels are more likely to be independent. However, those spatial patterns in Fig 4 that span multiple resolution elements in the beamformer reconstruction [40] will gain an advantage in SNR. Finally, we note that, although the ICA approach has many advantages, it remains limited, in part, because whilst some of the identified independent components will be of interest, others will necessarily reflect noise. A judgement must therefore be made as to which components to keep and which components to ignore (note that this is not a limitation of the RVE/ICA method *per se*, but rather is a general problem with ICA applied to any dataset). Here, the components we chose to keep were selected based on their spatial signature and task induced change in their timecourse.

In this paper, we chose to employ beamforming as a means to project extra-cranial data into brain space. Beamforming is a popular MEG inverse solution which exhibits good spatial resolution. Its adaptive nature also allows for excellent interference rejection properties, meaning that non-neuronal signals are minimised [38]. This said, a disadvantage is that spatially separate but temporally correlated neural sources are suppressed and often mislocalised. It is conceivable that, in some studies, researchers may find other inverse projection algorithms more suitable. We therefore note that entropic transformation and subsequent ICA could be used in conjunction with *any* source localisation methodology.

Results in Fig 4 show that spatially distinct regions generate independent entropic profiles. The fact that ICA yields regions that are well characterised in terms of their functional anatomy implies that entropy is a physiologically meaningful signal. This is further supported by the trial averaged entropy signals in Fig 5, which show that both the RM and Sternberg tasks evoke transient changes in entropy. In general, an expected increase in cortical processing (for example in the visual cortex during visual stimulus presentation) is characterised by increased entropy; this supports the idea that increased information processing necessitates the breakdown of synchrony to increase efficacy of encoding. Comparison of the timecourses of local entropy with the time frequency spectrograms in Fig 5 implies that task induced increases in entropy are temporally coincident with, and to an extent driven by, decreased alpha and beta band oscillatory amplitude, and therefore loss of synchrony across neurons. We again stress that time-frequency spectra and entropy timecourses are generated from the same underlying data. It is known that changing the oscillatory dynamics in a signal will alter entropy, with a prevalence of narrow band oscillations reflecting low entropy and broad band disordered signals reflecting high entropy. A negative relationship between oscillations and entropy should therefore be expected, however it is not the case that entropy is a surrogate for alpha and beta envelope signals.

In the brain regions we examine (selected on the basis of entropy—not oscillations), the amplitude envelope of low frequency (1 to 40Hz) oscillations is negatively correlated with the entropy timecourses as expected, whereas the envelope of higher (>50Hz) frequency oscillations is positively correlated. Importantly, this relationship differs between brain regions; for example, correlations between entropy and oscillatory amplitude in the visual cortex differed from those in the motor cortex, and bilateral insula cortex based on both spectral signature and correlation strength. It therefore follows that no simple one-to-one relationship exists between oscillations and entropy. It is also of interest to note the weaker negative correlation between delta and theta oscillations and entropy, compared to alpha and beta oscillations. Previous work [41] shows that theta oscillations are associated with working memory, and as prevalent narrow band oscillations would be expected to generate a decreased entropic signal in the same way as alpha and beta oscillations do. However, theta oscillations are task induced and often transient. Recall that the relationship shown in Fig 6 represents correlation between oscillatory envelope and entropy across the entire experiment; the theta oscillations themselves may therefore be dominated by other, more temporally sustained effects in alpha and beta bands; this may explain the weaker correlation.

In summary, entropy is not a direct reflection of oscillations, but rather a broadband composite signal that provides a broad picture of brain activity. It is driven, in part, by oscillations and allows a principled combination of frequency bands which differs depending on the brain region studied. This integration over frequency, coupled with the leaky integrator (collapsing measurements over time) means that entropy signals exhibit a high SNR. It is instructive to note that a change in oscillatory dynamics does not necessarily imply a change in entropy. For example consider a case where a stimulus evokes a shift from narrow band low frequency oscillations to narrow band high frequency oscillations, both signals are low entropy, and therefore may not generate a measured entropy change. Conversely, a change in entropy may not be observed by time-frequency decomposition: for example consider a case where a change in entropy is brought about by a subtle increase in broad band signal. This would generate a large change in entropy but may be ‘washed out’ in a time-frequency spectrogram which would be dominated by oscillations. We therefore suggest that oscillations and entropy should be treated as related, but potentially complementary metrics of electrophysiological brain activity. This complementarity is also evident from a neuroscientific standpoint. In the present manuscript we have stressed the importance of increased entropy in local networks in order to promote information processing. However healthy brain function must rely on a delicate balance between disorder and coordination between spatially remote cortical circuits (which facilitates integration of information). Long distance communication is likely to be mediated by neural oscillations [42], with different frequencies relating to different scales of cortical integration [41]. This means that considering both entropic and oscillatory transforms of the MEG signal has potential to provide insights into local activity, long range communication, and their relationship.

Finally, it is interesting to note a similarity between Fig 6, and measurements showing the relationship between neural oscillations and the blood oxygenation level dependent (BOLD) haemodynamic response measured using fMRI [3]. Temporal correlation between BOLD and oscillatory signals also shows a biphasic response, with negative correlation in the alpha and beta bands, and positive correlation at high frequency. Future work may therefore probe a potential relationship between entropy and BOLD.

### ‘Disorder’ in schizophrenia

An emerging picture of schizophrenia has implied that abnormal effective connectivity persists between separate nodes of the salience network, implying a reduction in integrative processing across this well characterised network [12]. In the present paper, we aimed to test the hypothesis that entropic measurement in schizophrenia would show increased disorder within separate nodes of this same network, reflecting, albeit indirectly, the breakdown of connectivity. Our findings support this hypothesis. Firstly, as expected from the disconnection hypothesis and in support of previous studies [14,15,16,17,18,19,20,21], we elucidate a general finding of increased task induced entropy change in patients relative to controls. Moreover, these abnormalities were observed to be statistically significant in the cingulo-insula regions as well as bilateral insula cortices and the right fronto-parietal network. Note that we tested all 12 regions highlighted in Fig 4, employing a multiple comparison test across regions; the fact that three of the four regions (cingulo-insula, left and right insula) that exhibited a significant result coincide spatially with the well-known salience network nodes offers support that this network may be abnormal in schizophrenia. (From a methodological point of view, this also highlights the importance of spatial localisation.) Our results support the recent finding suggesting a breakdown in salience network connectivity, and further add to this picture by suggesting that disrupted connectivity is accompanied by a concomitant increase in local entropy at each network node. We speculate that such increases might reflect ‘inefficient processing’ where more than the required degree of variation in the signal is necessary for carrying out specialised regional brain functions. In the presence of normal connectivity, such increase in variance would increase performance, but in schizophrenia this may only help to achieve near-normal performance in cognitive tasks. Note that, in addition to the salience network, we also observed significantly increased entropy in the right fronto-parietal network. The fronto-parietal network that we observe is a good spatial match to the dorsal attention network (DAN) that has been previously observed in fMRI. The DAN is known to exhibit an increased BOLD response during performance of cognitively demanding tasks and is thought to relate to both attentional and cognitive processes. The finding of abnormal entropy within this network is therefore plausible and this should be the topic of future study.

Our study also generated other findings. Firstly, the differences between schizophrenia patients and control subjects were found to change in time. In the RM task, the largest difference between patients and controls occurs at ~1s latency following a relevant stimulus. In the Sternberg task, the magnitude of the difference peaks within the 8–10s time window centred on presentation of the probe stimulus. In addition, temporally specific trends for higher entropy in schizophrenia were observed in other brain areas, for example in the left motor cortex entropy was slower to recover to baseline following a response in patients compared to controls. This highlights the importance of studying time resolved entropy, as distinct from single measures collapsed across task phases or whole experiments. Secondly, our results using MSE were in agreement with our results using RVE in showing increased task related change in entropy in patients relative to controls. However, we showed that temporal scale of the entropy measurement affected the results, with significant differences between patients and controls only observable at the shortest timescales. This implies that whilst assessment of entropy can provide insight into the neuro-pathological mechanisms underlying schizophrenia, those abnormalities only exist over short timescales.

Finally, we should consider the limitations of the present study in terms of the schizophrenia findings. The present work was designed to provide a proof of principle that our methodology could generate useful neurobiological insight into schizophrenia, but is not intended as a complete and exhaustive study. We present plausible results that support a hypothesis of disconnections in the salience network leading to increased local entropy in salience network nodes. However, the study was carried out in a small number of participants. Further, the patients involved were undergoing treatment and were therefore medicated. Previous work has shown that entropy effects are dependent on medication, age and symptomology. Specifically, young, highly symptomatic and medication naive patients are most likely to exhibit increased entropy. For this reason, whilst we demonstrate promise for the entropy measurement, further investigation in a larger group of patients is required.

## Conclusion

We have described a novel methodology, applying a combination of beamforming, rank vector entropy, independent component analysis and multi-scale-entropy, to MEG data in order to characterise brain activity in healthy controls and patients with schizophrenia. We have shown the utility of this technique in providing a broad spatio-temporal picture of brain activity and how it is modulated during cognitive tasks, with an increase in local neural processing characterised by a local and transient increase in entropy within the neural network. We have provided a demonstration of the clinical utility of our method, with our results confirming our hypothesis that schizophrenia is characterised by increased disorder in nodes of the salience network, supporting the idea that impaired connectivity between these nodes may contribute to core symptoms of the disease. The novel methodology in this manuscript, together with the results generated from it, has significant potential for application in future MEG measurements of neural signalling in health and disease. RVE is an as yet under-used technique, which exhibits extremely high SNR and offers novel insights into basic neuroscience. Additionally, the ease of use and non-invasive nature of the technique make it highly suited to clinical diagnosis and tracking efficacy of intervention. Although we demonstrate our method in schizophrenia, we believe that these methods could find application across a range of neurological disorders.

## Supporting Information

Data supporting our paper are supplied; these data comprise the raw images presented in Fig 4 of the main manuscript, as well as regional timecourses for the visual area, left motor area and the cingulo-insula regions. In accordance with the PLoS style, we provide a legend for these files below.

S1 Appendix Text and figures for the appendix to the main manuscript. (DOCX)

S1 Con Zip file containing data from the 1^st^ control subject. (ZIP)

S2 Con Zip file containing data from the 2^nd^ control subject. (ZIP)

S3 Con Zip file containing data from the 3^rd^ control subject. (ZIP)

S4 Con Zip file containing data from the 4^th^ control subject. (ZIP)

S5 Con Zip file containing data from the 5^th^ control subject. (ZIP)

S6 Con Zip file containing data from the 6^th^ control subject. (ZIP)

S7 Con Zip file containing data from the 7^th^ control subject. (ZIP)

S8 Con Zip file containing data from the 8^th^ control subject. (ZIP)

S9 Con Zip file containing data from the 9^th^ control subject. (ZIP)

S10 Con Zip file containing data from the 1^0th^ control subject. (ZIP)

S11 Con Zip file containing data from the 11^th^ control subject. (ZIP)

S1 Pat Zip file containing data from the 1^st^ patient. (ZIP)

S2 Pat Zip file containing data from the 2^nd^ patient. (ZIP)

S3 Pat Zip file containing data from the 3^rd^ patient. (ZIP)

S4 Pat Zip file containing data from the 4^th^ patient. (ZIP)

S5 Pat Zip file containing data from the 5^th^ patient. (ZIP)

S6 Pat Zip file containing data from the 6^th^ patient. (ZIP)

S7 Pat Zip file containing data from the 7^th^ patient. (ZIP)

S8 Pat Zip file containing data from the 8^th^ patient. (ZIP)

S9 Pat Zip file containing data from the 9^th^ patient. (ZIP)

S10 Pat Zip file containing data from the 10^th^ patient. (ZIP)

S11 Pat Zip file containing data from the 11^th^ patient. (ZIP)

S1 RVE Regional Images Images shown in Fig 4 of the main manuscript in .nii format. (NII)
Each .zip folder contains four files: Regional_timecourses_Relmod_*.mat contains an N x 3 matrix where the 3 columns contain the raw beamformer projected timecourses collapsed across the three representative regions for the relevance modulation dataset. (Note N represents number of time points). Regional_timecourses_Stern_*.mat contains an N x 3 matrix where the 3 columns contain the raw beamformer projected timecourses collapsed across the three representative regions for the Sternberg dataset. SternbergMarkerFile.mat contains details of the timing of the Sternberg task RelModMarkerFile.mat: Contains details of the timing of the relevance Modulation task.
