# Supplementary material for: Complexity Measures in Magnetoencephalography: Measuring "Disorder" in Schizophrenia
Source: PLoS One. 2015 Apr 17;10(4):e0120991. doi: 10.1371/journal.pone.0120991 (PMC4401778; doi:10.1371/journal.pone.0120991)
Supplement: S1 Appendix — (DOCX) [file pone.0120991.s001.docx]

**APPENDIX: Comparing spatial signatures in patients and controls:**

In this paper, it was not our aim to examine differences in spatial patterns between schizophrenia patients and healthy controls. Rather, the ICA methodology used to generate the spatial maps in Figure 4 was employed primarily as a means to reduce data dimensionality. As noted above, reducing several thousand voxels to a small number of independent components has multiple advantages, in particular by allowing simplification of data analysis. This said, to generate Figure 4, data were concatenated across all subjects (patients and controls) and it is conceivable that some spatial differences may exist between groups. There are two mechanisms by which such spatial differentiation might occur:

1. **Beamforming:** The brain areas identified in Figure 4 largely comprise individual uni-modal regions, e.g. left motor cortex, right motor cortex, etc. The ability to separate these regions reflects primarily the power of our source localisation algorithm to spatially isolate magnetic field topographies. With this in mind, the regions identified in Figure 4 relate principally to the mechanics of beamforming. The beamformer itself is data driven (via the data covariance matrix) and so if fundamental differences between MEG measurements in patients and controls exist, then this may alter beamformer spatial specificity, hence the spatial patterns in Figure 4.
2. **Connectivity breakdown:** Some of the spatial maps in Figure 4 identify multifocal networks – for example the left and right fronto-parietal networks. Such patterns necessarily require temporal correlation between entropy timecourses within each focal region. In this way the methodology can be thought of as reflecting a non-linear form of functional connectivity. If, for example, a total breakdown in functional connectivity was expected, then one might expect a difference in the spatial patterns identified,

Given these potential differences, a statistical analysis was undertaken in order to assess the degree to which patients and controls generate the same underlying spatial patterns when ICA is applied to entropy timecourses.

It was reasoned that, if patients and controls generated fundamentally different spatial signatures, then those signatures generated using only controls, and only patients, would lie in opposing tails of a null distribution which could be formulated by a random mix of participants. This was tested empirically using a Monte-Carlo approach. Firstly, separate datasets were made using the 23 available patient datasets, and 23 randomly selected control datasets. Beamforming, entropic transformation and ICA were applied, as described above, to generate spatial maps in these two cohorts independently. Following this, the resulting two sets of spatial maps, for patients and controls, were compared quantitatively with those generated by inclusion of all individuals in the concatenated dataset (i.e. those spatial patterns shown in Figure 4, henceforth termed the concatenated maps). Quantification was achieved via assessment of variance explained in the concatenated maps by the subset maps; this was computed for each individual map separately, and an average across the whole set of 12 maps was also calculated. Following this, equivalent calculations were performed but, rather than including only patients, or only controls, 23 datasets were picked at random. Once again the similarity of these pseudo-group spatial maps with the concatenated maps was quantified. If a significant difference between patients and controls, in terms of spatial component signatures, was to be found then this would require variance explained by the patient or control groups to fall outside the empirical null distribution.

On average, the concatenated maps, and control only maps were 88 ± 14% correlated. This is compared to an 84 ± 13% correlation between the concatenated maps and the patient only maps. This high correlation suggests that the concatenated maps observed in Figure 4 represent spatial signatures that can be found in patients and controls. To test this result statistically, the empirical null distributions are shown in Figure 9. Variance explained using control only data (yellow) and patient only data (red) is also shown. Figure 9A shows the results for each spatial map in Figure 4 separately; Figure 9B shows the average result across all maps. Note first that no significant spatial difference between patients and controls was observed, either in the group average, or in the individual networks. Note also the robustness of the spatial maps presented in Figure 4. The concatenated maps are generated using 53 datasets, whereas for all data contributing to the null distribution, only 23 of those 53 datasets have been employed. Despite this, the null distribution shows, on average, an 87% spatial correlation with the concatenated maps. This robustness, coupled with the lack of significant difference between patients and controls, implies that the brain regions identified in Figure 4 can be considered as a set canonical spatial topographies that are likely representative of the spatial specificity of the MEG/Beamformer/Entropy/ICA methodology, and not a feature of subjects, or subject group classification.


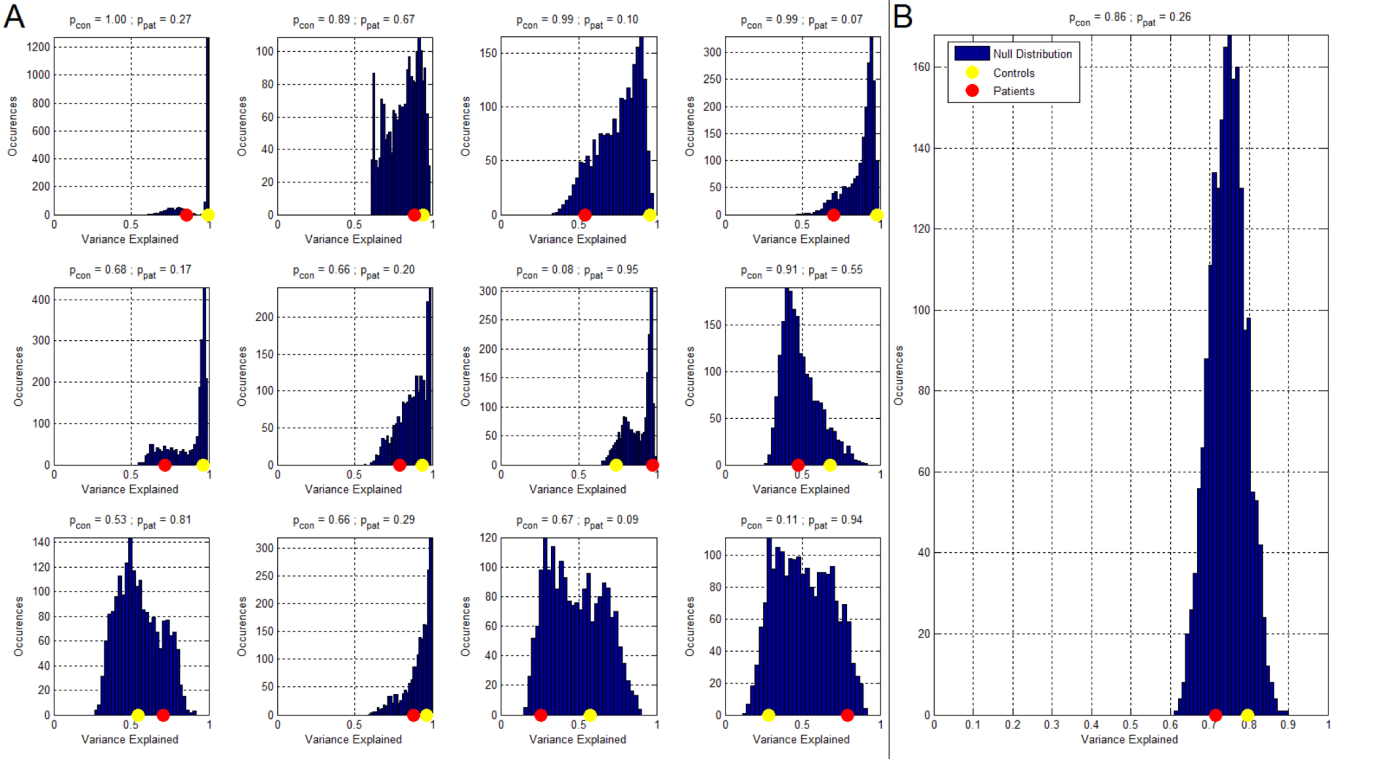


*Figure 9: Spatial agreement between patients only, controls only, and the concatenated dataset. Variance explained in the concatenated dataset by patients only is shown by the red markers. Variance explained in the concatenated dataset by controls only is shown by the yellow markers. The blue histogram shows a null distribution in which patients and control datasets are selected randomly. A) Shows the case for each network in Figure 4 individually. B) shows the average result across all networks. Note that on average, neither patients nor controls differ significantly from the null distribution in terms of spatial similarity.*
